# Short- and long-term outcome of allogeneic stem cell transplantation in infants: A single-center experience over 20 years

**DOI:** 10.3389/fped.2022.956108

**Published:** 2022-08-22

**Authors:** Justyna Miśkiewicz-Bujna, Izabella Miśkiewicz-Migoń, Zofia Szmit, Dawid Przystupski, Monika Rosa, Anna Król, Krzysztof Kałwak, Marek Ussowicz, Ewa Gorczyńska

**Affiliations:** ^1^Department of Pediatric Bone Marrow Transplantation, Oncology, and Hematology, Wroclaw Medical University, Wroclaw, Poland; ^2^Department of Paediatric Anaesthesiology and Intensive Care, Wroclaw Medical University, Wroclaw, Poland

**Keywords:** allogeneic, hematopoietic (stem) cell transplantation (HCT), infant-age, neurological complications, sequelae

## Abstract

**Introduction:**

Allogeneic hematopoietic stem cell transplantation (allo-HSCT) is a treatment method for a wide range of malignant and non-malignant diseases. Infants constitute a distinct patient group, especially due to their organ immaturity and differences in drug metabolism. The present paper aims to analyse the short- and long-term outcomes after allo-HSCT in infants.

**Material and methods:**

In the study period, 67 patients under 12 months of age underwent allo-HSCT. This study is a retrospective analysis of patient medical records, in the form of paper and electronic documentation.

**Results:**

The probability of 5-year OS was 69% and 72% in patients with malignant and non-malignant diseases, respectively. The allo-HSCT from a matched donor was associated with improved OS in comparison to haploidentical donor (0.8 vs. 0.58%, *p* = 0.0425). The overall incidence of acute graft-vs.-host disease (aGVHD) was 59.3%, and grade III–IV aGVHD was diagnosed in 23% of patients. The 100-day non-relapse mortality (NRM) in the study cohort was 17.9%, while the 5-year NRM was 26.9%. Among the causes of NRM, infections occurred in 83.3% of patients, and aGVHD in 16.3% of individuals. Twenty-two children (32.8%) required hospitalization in the pediatric intensive care unit (PICU). The median length of PICU hospitalization was 6 days (range 1 to 12 days). Late sequelae diagnosed during post-transplant surveillance included ocular disorders in 26.8% of patients, cardiac complications in 4.4%, as well as endocrinopathy with short stature (<3rd percentile) in 37.2% and overt hypothyroidism in 35.4%. In the long-term perspective, 83.3% of survivors were able to attend a regular school.

**Conclusions:**

Improvements in unrelated donor availability, and better supportive care resulted in better outcomes. Management of infant allo-HSCT recipients requires the formation of multi-disciplinary specialist teams. In addition, the role of parental empowerment must be acknowledged; for example, in speech therapy and rehabilitation.

## Introduction

Allogeneic hematopoietic stem cell transplantation (allo-HSCT) is a treatment method for a wide range of malignant and non-malignant diseases. This procedure is widely performed in both children and adults; however, in the youngest group of patients (i.e., those <1 year of age), the data are scarce and mainly concern the wider pediatric group (1–7). Despite the curative potential of allo-HSCT, this procedure has been associated with numerous early and late complications. Many factors affect the outcome of allo-HSCT, such as the age of the recipient, type of donor, degree of human leukocyte antigen (HLA) matching, conditioning regimen, stem cell source, supportive therapy, and immunosuppressive treatment (8, 9). Adverse effects occurring before the day 100 after HSCT are classified as early complications, which include conditioning-related toxicity (e.g., mucositis), endothelial complications (e.g., sinusoidal obstruction syndrome [SOS], transplant-associated microangiopathy), acute graft-vs.-host disease (aGVHD), severe opportunistic infections (bacterial, viral, and/or fungal), and multi-organ failure (7, 10). In infants, due to their lower organ maturity and limited compensative abilities, early complications are more likely to cause destabilization of the patient's condition, thus requiring intensive care, compared to older patients. Late complications—that is, those diagnosed at least 100 days after allo-HSCT—include chronic graft-vs.-host disease (cGVHD), as well as long-term sequelae such as cardiovascular diseases, osteoporosis, endocrinopathy, ocular disorders, and neurological disturbances (10, 11). Infants constitute a distinct patient group, due to their organ immaturity and differences in drug metabolism, and post-transplant toxicities may be higher in infants than in older children. Another distinctive feature in this age group is high prevalence of inborn errors of immunity or metabolism, that are responsible for allo-HSCT referral. The aim of this study is to analyse the short- and long-term complications and transplant outcomes in infants undergoing allo-HSCT.

## Patients and methods

This study is a single-center retrospective analysis of 67 patients who underwent allo-HSCT before the age of 365 days in the Department of Pediatric Hematology, Oncology and Bone Marrow Transplantation in Wroclaw within the period 1999–2019. This study is a retrospective analysis of data collected from patient medical records in the form of paper and electronic documentation, and databases created based on these medical records. The patient and transplant characteristics are provided in Table 1. Patients were prepared with myeloablative conditioning protocols (MAC, based on equivalent of 16 mg/kg BW of busulfan) or with reduced-intensity (RIC)/reduced-toxicity (RTC) regimens containing combinations of treosulfan with fludarabine, melphalan, or thiotepa (12, 13). In two patients minimal, non-myeloablative (NMA) conditioning was administered. Patients undergoing transplantation were screened weekly for the replication of viruses [adenovirus (ADV), polyoma BK virus (BKV), human cytomegalovirus (CMV), Epstein-Barr virus (EBV)], blood cultures and multidrug resistant bacteria colonization as a part of routine monitoring [as summarized by Salamonowicz-Bodzioch et al. (14)]. However, until 2007 the diagnostics of ADV, BKV and EBV was limited in comparison to the later period. First-line intravenous antibiotic empiric therapy usually included a broad-spectrum beta-lactam and aminoglycoside. Antifungal prophylaxis with fluconazole (until 2008) or oral posaconazole (after 2008) was started at the beginning of the conditioning regimen and continued until discharge, but in patients treated for GVHD was continued until the end of immunosuppressive treatment. In the case of secondary prophylaxis for invasive fungal disease variable mold-active agents were administered. Prophylaxis for viral infections consisted of acyclovir from the beginning of the conditioning regimen to the end of immunosuppression. In the case of CMV replication, pre-emptive antiviral therapy with ganciclovir was started. In the case of symptomatic BKV- or ADV-hemorrhagic cystitis or ADV replication with or without clinical manifestations, intravenous cidofovir with oral probenecid was used once a week. If EBV-DNA in the blood was positive (≥5,000 copies/mL), rituximab (375 mg/m^2^) was administered weekly until replication was negative. Prophylactic trimethoprim/sulfamethoxazole was given to all patients every day before and 3 times/week on consecutive days until at least 1 month after the end of immunosuppression.

**Table 1:** Patient and transplantation characteristics.

	**Matched donor**		**Haploidentical donor**		**CBT**	
	***n***	**%**	***n***	**%**	***n***	**%**
Number of patients (*n*)	35	52.24	26	38.81	6	8.96
Patient age, days median (range)	239 (98–365)		235 (60–347)		273 (53–352)	
**Gender**						
Female	10	28.57	5	19.23	2	33.33
Male	25	71.43	21	80.77	4	66.67
**Type of donor**						
Matched unrelated	30	85.71	0	0	5	83.33
Matched sibling	5	14.29	0	0	1	16.67
Partially matched parental	0	0	26	100.0	0	0
**Stem cell source**						
Bone marrow	7	20.0	0	0	0	0
Peripheral blood	28	80.0	26	100	0	0
Cord blood	0	0	0	0	6	100.0
**CMV status**						
Positive recipient/positive donor	10	28.57	2	7.69	1	16.67
Negative recipient/negative donor	14	40.0	5	19.23	3	50.0
Negative recipient/positive donor	6	17.14	1	3.85	1	16.67
Positive recipient/negative donor	4	11.43	2	7.69	1	16.67
Unknown	1	2.86	16	61.54	0	0
**Conditioning**						
Myeloablative conditioning (busulfan-based)	18	51.43	18	69.23	4	66.67
Reduced toxicity/reduced intensity conditioning (treosulfan-based)	15	42.86	8	30.77	2	33.33
Other (non-myeloablative conditioning)	2	5.71	0	0	0	0
**Primary diagnosis**						
**Non-malignant diseases**						
**Primary immunodeficiency**						
SCID	7	20.0	14	53.85	0	0
Omenn syndrome	3	8.57	6	23.08	1	16.67
Wiskott-Aldrich syndrome	5	14.29	0	0	1	16.67
Chronic granulomatous disease	2	5.71	0	0	0	0
Kostmann syndrome	1	2.86	0	0	1	16.67
NEMO deficiency	1	2.86	0	0	0	0
Hemophagocytic lymphohistiocytosis	1	2.86	0	0	0	0
**Inborn errors of metabolism**						
Hurler syndrome	0	0	0	0	2	33.33
Krabbe disease	1	2.86	0	0	0	0
Sandhoff Disease	0	0	0	0	1	16.67
X-linked adrenoleukodystrophy	1	2.86	0	0	0	0
**Other**						
Osteopetrosis	1	2.86	1	3.85	0	0
Diamond-Blackfan anemia	1	2.86	0	0	0	0
Severe aplastic anemia	1	2.86	0	0	0	0
**Malignant diseases**						
Acute lymphoblastic leukemia	5	14.29	0	0	0	0
Acute myeloid leukemia	3	8.57	2	7.69	0	0
Juvenile myelomonocytic leukemia	2	5.71	3	11.54	0	0
**Year of transplantation**						
1999–2008	2	5.71	20	76.92	0	0
2009–2019	33	94.29	6	23.07	6	100.0

The symptoms and severity of acute and chronic GVHD were assessed according to the modified Glucksberg classification and the Seattle Criteria, respectively (15, 16).

The assessment of long-term sequelae included incidence and organ manifestations in children surviving beyond 2 years from allo-HSCT. Patients were screened, at least once per year, with a liver function test (alkaline phosphatase, alanine transaminase, aspartate transaminase, serum bilirubin, serum albumin, lactate dehydrogenase), serum ferritin, renal function tests (serum creatinine, blood urea nitrogen, magnesium), blood pressure, endocrine function tests (thyroid-stimulating hormone, triiodothyronine, thyroxine, cortisol, adrenocorticotropic hormone, luteinizing hormone, follicle stimulating hormone, measurement of height and weight), and calcifediol/vitamin D blood level. Developmental surveillance was evaluated according to the Polish guidelines using developmental milestones and skills attainments (17). Dental and ophthalmologist examination, abdominal ultrasound, ultrasound of the ovaries or testes, as well as bone density scan were performed once per 1–2 years. Referral for audiological diagnostics was made at physician discretion in patients showing speech delay or hearing impairment. The patients or legal guardians gave their written informed consent for the analysis of clinical data. Ethical approval was waived by the local Ethics Committee of Wroclaw Medical University, in view of the retrospective nature of the study and because all procedures were performed as part of routine care.

### Statistical analysis

The primary endpoints were overall survival (OS), defined as the time from allo-HSCT to death or the last report from patients with no events; event free survival (EFS) defined as the time from allo-HSCT to death, graft failure or relapse; and non-relapse mortality (NRM), defined as a death that occurred in the absence of disease relapse or progression or within 28 days of transplant. Secondary endpoints were graft failure, incidence of relapse, aGVHD, cGVHD, and pediatric intensive care unit (PICU) admissions. Survival curves were estimated using the Kaplan–Meier method and comparison between cohorts was carried out by log-rank test. Differences for categorical data were studied using a two-tailed Fisher's exact test. Statistical analyses were conducted using the Statistica 13 software (TIBCO Software Inc. 2017, STATISTICA, version 13, Dell, OK, USA) and R ver. 4.1.1.

## Results

In the analyzed period, 67 patients below 12 months of age underwent allo-HSCT, which comprised 7.6% of all procedures. Fifty two out of 67 patients (77%) were transplanted due to non-malignant diseases. All infants received non-irradiation containing conditioning protocols, that were classified as MAC (busulfan-based), RTC/RIC (treosulfan-based) or NMA protocols. Median follow-up was 1,373 days (range 3–7875 days). The survival results for the study cohort are presented in Table 2. Five patients died before engraftment on days +3, +5, +6, +13, and +31, due to sepsis. The median time for achieving a sustained neutrophil count over 500/μl was 13 days (range 9–24 days). The median time to platelet count over 20,000/μl was 12 days (range 6–42 days) and over 50,000/μl was 15 days (range 8–57 days). Primary graft failure occurred in 2 infants and secondary graft failure in nine patients; nine of these patients required secondary allo-HSCT and two of them died before re-transplantation due to infections. Relapse of malignant disease was diagnosed in four patients, three of which underwent a second allo-HSCT. Twelve children underwent a second transplantation, with the median time for this procedure being 137 days (range 29–1,610 days), due to graft failure or disease relapse. Ten patients are alive after the second allo-HSCT due to primary graft failure (1 pt), secondary graft failure (7 pt), or relapse (2 pt), and two patients re-transplanted due to relapse (1 pt) or primary graft failure (1 pt) succumbed due to disease.

**Table 2:** Impact of risk factors on the outcome and incidence of posttransplant complications.

**Probability**		**Total (1999–2019)**	**Year of transplantation**			**Primary disease**			**Conditioning protocol**			**Donor type**		
			**1999–2008 (*n* = 22)**	**2009–2019 (*n* = 45)**	***p*-value**	**Malignant (*n* = 15)**	**Non-malignant (*n* = 52)**	***p*-** **value**	**Myeloablative (*n* = 40)**	**RTC/RIC (*n* = 25)**	**p-value**	**Matched (*n* = 41)**	**Haploidentical (*n* = 26)**	***p*-value**
OS		0.72	0.59	0.78	0.095	0.73	0.71	0.8645	0.65	0.80	0.1869	0.80	0.58	0.0425*
EFS		0.52	0.41	0.58	0.1063	0.47	0.54	0.6029	0.48	0.56	0.4527	0.59	0.42	0.1263
NRM		0.27	0.36	0.22	0.2504	0.20	0.29	0.7422	0.33	0.20	0.3942	0.20	0.38	0.10
Graft-failure incidence		0.16	0.27	0.11	0.1572	0.13	0.17	1.0000	0.20	0.12	0.5087	0.12	0.23	0.3150
Second allo-HSCT incidence		0.18	0.27	0.13	0.1874	0.27	0.15	0.4439	0.20	0.16	0.7541	0.15	0.23	0.5152
Relapse incidence (in malignant diseases)		0.27	0.17	0.33	0.6044	0.27	n/a	-	0.25	0.33	1.0	0.30	0.20	1.0
aGVHD incidence	No	0.41	0.45	0.39	0.5959	0.33	0.43	0.7659	0.47	0.29	0.1882	0.35	0.50	0.3065
	Any (I-IV)	0.59	0.55	0.61		0.67	0.57		0.53	0.71		0.65	0.50	
	Severe (III-IV)	0.23	0.20	0.25	0.7615	0.40	0.18	0.1655	0.24	0.24	1.0	0.30	0.13	0.1318
cGVHD incidence	No	0.57	0.40	0.64	0.1329	0.50	0.60	0.7469	0.50	0.64	0.4137	0.61	0.50	0.4426
	Any (mild+moderate+severe)	0.43	0.60	0.36		0.50	0.40		0.50	0.36		0.39	0.50	
	Moderate+severe	0.26	0.60	0.15	0.0017*	0.42	0.21	0.1559	0.37	0.14	0.0608	0.17	0.44	0.0219*
Post-transplantation BKV replication incidence^‡^		0.24	0.05	0.33	n/a	0.40	0.19	n/a	0.25	0.24	n/a	0.34	0.08	n/a
Post-transplantation ADV replication incidence^‡^		0.31	0.05	0.44	n/a	0.33	0.31	n/a	0.25	0.44	n/a	0.44	0.12	n/a
Post-transplantation CMV replication incidence		0.35	0.23	0.41	1.0	0.20	0.38	0.2252	0.25	0.50	0.0661	0.33	0.38	0.6058
Post-transplantation EBV replication incidence^‡^		0.25	0.05	0.36	n/a	0.40	0.21	n/a	0.20	0.32	n/a	0.39	0.04	n/a
SOS/VOD incidence		0.13	0.09	0.16	0.7066	0.27	0.10	0.1048	0.23	0.00	0.01*	0.17	0.08	0.4646

*n/a, not applicable.*

P-values <0.05 are marked with asterisk (*).

*^‡^Patients treated in years 1999–2008 were underdiagnosed for ADV, BKV and EBV infections. Data not evaluated due to measurement bias*.

Death occurred in 19 (28.3%) children, in one patient as a consequence of juvenile myelomonocytic leukemia relapse and in 18 patients due to NRM. The probability of death in children with malignant and non-malignant diseases was 26.7 and 28.8%, respectively. The interval from allo-HSCT to death was 69 days (range +3 to +341 days). The 100-day NRM in the studied cohort was 17.91% and the 5-year NRM was 26.86%. The NRM was not affected by transplantation period, primary disease, conditioning protocol, and donor type. The NRM causes included infections (bacterial, 7; CMV, 5; other, 3) in 15 (83.3%) patients, and GVHD in 3 (16.3%) individuals. The incidence of confirmed viral infections is presented in respective subgroups, but the analysis was not possible due to measurement bias.

**Figure 1:**
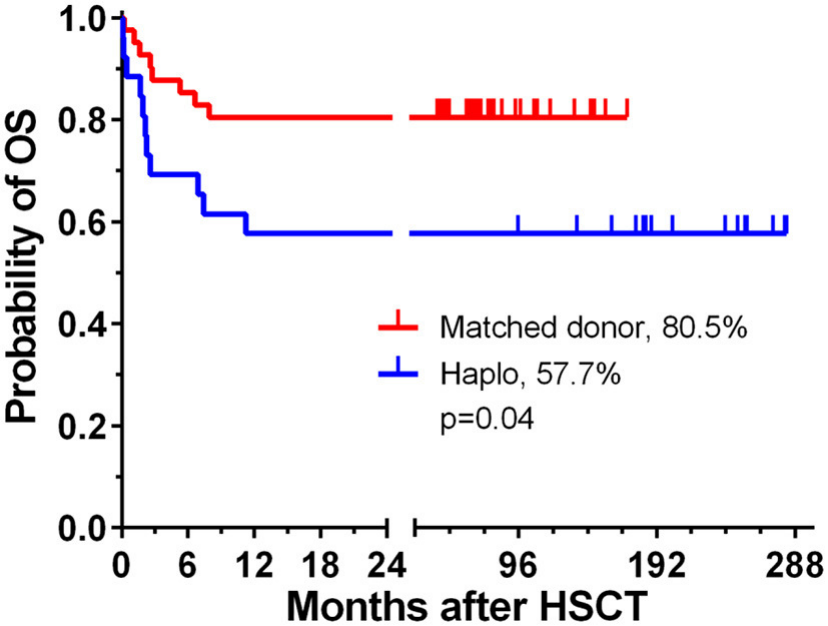
Overall survival of children transplanted from matched related or unrelated donors (Matched), and haploidentical (Haplo) donors.

The probability of 5-year OS was 73 and 71% in patients with malignant and non-malignant diseases, respectively; however, the difference was not statistically significant. The allo-HSCT from a matched donor was associated with improved OS in comparison to haploidentical donor (0.8 vs. 0.58%, *p* = 0.0425, Figure 1). In relation to the stem cell source, the probability of OS was compared among patients transplanted with non-manipulated material from matched related or unrelated donors, T-cell depleted haploidentical donors, or with cord blood, but the difference was not statistically significant (Supplementary Figures 1, 2). The probability of OS in patients transplanted in years 1999–2008 and 2009–2019 was 59 and 78%, respectively, but the difference was not significant (Supplementary Figure 3). Sixty-four engrafted patients were evaluated for symptoms of acute GVHD. The overall incidence of aGVHD was 59.4%, and grade III–IV aGVHD was diagnosed in 23% of patients. Chronic GVHD was assessed in 54 patients who survived beyond day +100. The overall incidence of cGVHD was 42.6%, and the grade was moderate to severe in 25.9% of patients. There was no statistical significance between CMV positive and negative recipients who developed cGVHD (*p* = 0.7576), as well as between patients transplanted from an MSD and alternative donors (*p* = 0.0644). Twenty-two (32.8%) children required hospitalization in the pediatric intensive care unit due to respiratory or circulatory failure and need for mechanical ventilation in all cases. The median time between allo-HSCT and PICU admission was 36 days (range +0 to +506 days). The median length of PICU hospitalization was 6 days (range 1 to 12 days). Indications for PICU admission are listed in detail in Table 3. Thirteen children died during PICU hospitalization, and 9 survived and are alive, but only three children do not exhibit significant developmental disabilities. Comparison between patients transplanted in years 1999–2008 and 2009–2019 did not reveal any risk factors (Supplementary Table 3). Analysis of *ex vivo* haploidentical graft engineering methods did not show differences between subgroups undergoing the immunomagnetic CD34 enrichment or T-cell depletion (Supplementary Table 4).

**Table 3:** Indications for PICU admission.

**Causes of hospitalization in the pediatric intensive care unit after HSCT**	**Number of patients (*n* = 67)**
Respiratory or cardiorespiratory failure	13/67 (19%)
Sepsis	4/67 (6%)
Neurological incident	2/67 (3%)
Acute kidney injury	1/67 (1%)
Acute haemolysis	1/67 (1%)
Post-operative critical care	1/67 (1%)

### Long term sequelae

The impact of allo-HSCT on education was evaluated in 48 patients (19 excluded due to death). Forty individuals (83.3%) attended a regular school, two (4.2%) used individual teaching at home, five (10.4%) attended inclusive or specials schools, and one (2.1%) patient attended school a year later, due to delayed social development, and is currently studying at normal university. Long-term neurological sequelae are listed in detail in Table 4. Ocular disorders occurred in 18 (26.9%) of patients; the most common was vision defect defined as myopia, hyperopia, or astigmatism, which was diagnosed in 13 (19.4%) individuals, 12 of which needed visual aids and glasses. Three (4.5%) patients suffered from the dry-eye syndrome, two (3%) children had CMV infection, and the others had various problems such as corneal opacity, oculomotor muscle paralysis, and optic nerve atrophy. One patient also had horizontal nystagmus, while another suffered from concurrent strabismus due to progressive multi-focal leukoencephalopathy. The analysis of vision or hearing defects did not reveal any statistically significant risk factors (Supplementary Tables 1, 2). Cardiac complications were observed in three (4.5%) patients who developed cardiomyopathy, two of which required ACE inhibitors. Growth development was evaluated in 43 patients. Short stature (<3rd percentile) at the latest medical check-up was recorded in 16 (37.2%) patients. Two children received growth hormone therapy. Thyroid function was assessed in 48 patients. Seventeen children (35.4%) developed hypothyroidism requiring levothyroxine substitution, two (4.2%) had sub-clinical hypothyroidism, and seven (14.6%) had transient disorders not requiring medication. Pubertal development was evaluable in 17 patients who reached a minimum of 12 years of age; all of them had normal pubertal development, based on the judgment of the treating physician. No ovarian or testicular disorders were diagnosed during the follow-up, and no proven fertility impairment was observed.

**Table 4:** Long-term neurological sequelae.

**Neuropsychiatric disturbances**	**Number of patients (*n* = 48)**
Delayed motor skills	13/48 (27%)
Speech delay	11/48 (23%)
Seizures	3/48 (6%)
Epilepsy	2/48 (4%)

## Discussion

The infant cohort was characterized by distinct primary diagnoses, as well as high representation of primary immunodeficiencies and inborn errors of metabolism (77.6%). Among the malignant diseases, there was an evident prevalence of high-risk pediatric infant leukaemias, associated with unfavorable prognoses. A higher number of procedures were conducted in the years 2009–2019, compared to the first decade (1999–2009; 45 vs. 22); however, this was proportional to the total number of HSCTs in the transplant center and did not reflect changes in patient referral or indications for allo-HSCT. In the whole group, 28.4% of patients died after transplantation without differences between malignant and non-malignant diseases, proving that children transplanted before 12 months of age are more likely to suffer from such complications than those transplanted after finishing their first year (6). In the pre-engraftment period, high rates of PICU admissions (32%) and deaths due to sepsis (7.4%) were observed. This phenomenon highlights the elevated risk of allo-HSCT complications in the infant population (18). A mortality of 40.9% was reported before 2009, while 22.2% was observed during 2009–2019; the improvements in survival rates were similar to those in studies reporting only slow progress in this age group (1–4). Parikh et al. have evaluated a total of 2,498 infants transplanted over a period of 14 years, and reported gradual improvement in the outcomes associated to non-malignant diseases (*n* = 2026; 3-year overall survival: 2000–2004, 375/577 [65.0%]; 2005–2009, 503/699 [72.0%]; and 2010–2014, 555/750 [74.0%]) compared with malignant conditions, who had poorer survival rates and did not show improvement over time (3-year overall survival: 2000–2004, 109/199 [54.8%]; 2005–2009, 104/161 [64.6%]; and 2010–2014, 66/112 [58.9%]) (2). In this study, the observed phenomena of excessive toxicity and mortality were associated with multiple factors. The lack of a significant difference in probability of 5-year OS in patients with malignant and non-malignant diseases was due to the results in the first decade of the study, with a higher number of non-malignant patients undergoing haploidentical allo-HSCT, due to the limited availability of matched related or unrelated donors. Our analyses revealed that the choice of haploidentical donor was connected with a higher risk of death in children and the most striking difference between analyzed periods was the better availability of matched unrelated donors. The improvement in survival rates in the second decade was primarily the result of better matched unrelated donor availability, who replaced haploidentical donors and transplant procedures with CD34 enrichment. In the first decade, availability of unrelated donors was limited due to the fact, that the number of donors in BMDW (Bone Marrow Donors Worldwide) was around 1 million in 1998 and donor search and collection times were unacceptably long (19). In the first decade, only two out of 22 children were transplanted from matched donors, and 20/22 (90%) underwent the haploidentical transplantation, whereas haploidentical transplantations were performed in only 6/45 children (13%) in the second time period. The relatively low number of cord blood transplantations was a consequence of the center profile, with high experience in graft engineering and T-cell depleted stem cell transplantations and limited access to UCB, due to a lack of public cord-blood banking in Poland. In addition, anti-infective prophylaxis and therapy in the first decade was less effective due to limited access to mold-active antifungals (e.g., voriconazole, posaconazole, and liposomal amphotericin B) and antivirals (e.g., cidofovir). Other factors directly affecting the post-transplant toxicity were the intensity of conditioning and serotherapy. Patients transplanted before the year 2010 were more commonly treated with myeloablative conditioning protocols, including busulfan-based protocol, and only after that date were reduced toxicity regimens, including treosulfan-fludarabine backbone, used as a standard of care. One of the drawbacks of busulfan-based conditioning regimens is the incidence of sinusoidal obstruction syndrome/hepatic venoocclusive disease (SOS/VOD), with a mean incidence of 14%, which can be potentially fatal (20). In our cohort, concerning infants undergoing HSCT, the overall incidence of SOS was similar (13.4%) but all SOS/VOD developing patients had busulfan-based conditioning regimens. Changes in the diagnostic criteria for SOS/VOD and the wider availability of defibrotide for the treatment of this complication were other factors limiting the adverse effect in post-transplant outcomes. Viral infections constitute a significant cause of morbidity and mortality after HSCT, but the incidence of viral replication in the literature varies and has a wide range; depending, for example, on the type of disease (14, 21). We observed a statistically significant association between post-transplantation ADV, and EBV, replication and type of the donor, but this was a consequence of underdiagnosing in patients transplanted between 1999 and 2009, when the number of haploidentical donors was higher than in the second period. Improvement in viral diagnostics and use of cidofovir were introduced into clinical practice in years 2004–2007 (22–24). Among the non-infectious complications, graft-vs.-host disease is one of the most life-threatening and devastating complications. In our study, the incidence of aGVHD was 59.4%, while that of cGVHD was 42.6%. Hierlmeier et al. have observed a 59.5% incidence of aGVHD in older pediatric patients after allogenic HSCT (median age 7 years), similar to our results in infants undergoing allo-HSCT. On the other hand, their analyses revealed a lower incidence of cGVHD (22.2%) (6). This effect can be only partially explained by increased center and team experience in the second decade, especially with the progress of serotherapy for the prevention of non-infectious complications. The manipulations with T-lymphocyte add-backs and immunosuppression tapering were used in the first analyzed decade in case of decreasing donor chimerism, which resulted in GVHD induction. In addition, in the second time period the GVHD treatment was based on anti-cytokine therapy with etanercept and basiliximab, and extracorporeal photopheresis, which replaced high-dose (>2 mg/kg/day) methylprednisolone and posttransplant anti-thymocyte globulin treatment used earlier.

Even without a noticeable impact on the survival rates, the incidence of cGVHD had a significant impact on the long-term quality of life after allo-HSCT, and requires further studies in larger populations.

The effects of long-term sequelae on both survival and quality of life in conjunction have only been thoroughly studied in recent years (25). Pediatric HSCT recipients carry a significantly greater burden of morbidity, not only compared with non-cancer populations but also compared with conventionally treated cancer patients (26). The risk of toxicity may be particularly increased in patients in early developmental stages, due to the immaturity of drug excretion mechanisms and metabolism (27). Meanwhile, the immaturity of the central nervous system is responsible for neurological and developmental complications. In our cohort, delayed motor skills were recorded in 19% of patients, and speech delay in 16% of the children.

As has been showed by Shah et al. pediatric HSCT recipients can present neurocognitive deficiencies that are both acute and chronic. Although acute neurological deficits may occur and improve over time, progressive and permanent declines in neurocognitive function have been observed in many patients (28). Psychomotoric developmental deficits affect the quality of life of pediatric HSCT recipients and, due to their irreversible character, can by only prevented by the optimal choice of pre- and post-transplant pharmacotherapy and elimination of excessive toxicities. In addition to direct neurotoxic effects, neurocognitive development is affected by sensory impairment, especially worsened eyesight and hearing loss. In this study, we found that 26.9% of patients had ocular disorders, which was particularly devastating in terms of cognitive development due to the dominant role of vision in perception and learning. In the healthy peers, the incidence of refractive errors is estimated at 1.2%, and increased incidence of vision defects in the HSCT recipients can be caused by such risk factors as limited outdoor activity and limited light intensity (29). Problems with hearing were incidentally found in the study group, although their intensity was mild. It can be hypothesized, that diminished hearing acuity was a consequence of both pharmacotherapy with ototoxic drugs (such as aminoglycosides used for febrile neutropenia, furosemide, and cyclosporine A), and infections (such as CMV) and neurotoxicity of conditioning protocol.

Sensoric impairment not only reduces the quality of life, but also can cause problems with reading and learning. Furthermore, in contrast to neurotoxicity, such impairment can be reverted or effectively treated through optical correction or hearing aids. The endocrine function is impaired after HSCT, especially in patients undergoing axial radiotherapy/total body irradiation, but these procedures are not performed in children below the age of 1 year. However, the direct cytotoxic effect of conditioning protocol to the hypothalamic–pituitary axis and endocrine glands can result in endocrine failure. Endocrinopathy is one of the most devastating complications after HSCT in children, manifesting as growth impairment, disturbed bone mineralization, and delayed puberty (30). The consequences of hormonal failure can be partially reversed by early diagnostics based on regular monitoring of endocrine system function and substitution, such as growth hormone therapy, levothyroxine substitution, and sex hormone replacement therapy. In the light of the reduced gonadotoxic activity of treosulfan, this can be another argument in favor of eliminating busulfan-based conditioning regimens (31). The establishment of long-term patient surveillance is necessary in transplant survivors, as complications can may occur 30 to 40 years after HSCT, including cardiovascular complications, which are responsible for a 5–7 times increased risk of mortality, in comparison with healthy peers (32).

## Conclusions

Infants undergoing allo-HSCT present distinct clinical characteristics and exhibit more complex complication profiles than older children. In our opinion delaying the HSCT beyond the age of 1 year is not warranted for most patients, but outcome can be improved by modern conditioning protocols (use of RIC/RTC conditioniong, therapeutic drug monitoring) and better supportive care. Careful long-term monitoring, along with the prompt management of treatment-related late complications, may be crucial in this group of patients. Early diagnostics and the correction of reversible post-transplant complications are highly beneficial, in terms of quality of life, and can prevent the permanent disability of childhood HSCT survivors. The management of infant HSCT recipients requires the formation of multi-disciplinary specialist teams to offer the best medical care, where the specialists taking care of early childhood survivors must be educated with respect to the diagnostics for and therapy of long-term complications, due to the long life expectancy of their patients. The role of parental empowerment must also be acknowledged, in terms of the establishing of appropriate conditions for learning and reinforcement (e.g., speech therapy or rehabilitation), in order to improve the child's development. In addition, the transplantation outcome evaluation should not be restricted to the engraftment efficacy and survival analysis, but the long-term sequelae must also be scrutinized, which is particularly difficult due to patient loss. We acknowledge that the key limitation of our study is the small number of patents considered, but specific outcomes in this population pose challenges that cannot be overlooked and need to be addressed.

## Data availability statement

The datasets for this article are not publicly available due to concerns regarding participant/patient anonymity. Requests to access the datasets should be directed to the corresponding author via ussowicz@tlen.pl.

## Author contributions

Patient care, data collection, analysis, statistics, and manuscript preparation and acceptance: JM-B and IM-M. Data collection, analysis, and manuscript acceptance: ZS. Patient care, data collection, statistics, analysis, and manuscript acceptance: DP. Data collection, statistics, analysis, and manuscript acceptance: AK. Patient care, data collection, analysis, and manuscript acceptance: MR and KK. Concept, patient care, data collection, analysis, manuscript preparation, and final acceptance: EG and MU. All authors have read and agreed to the published version of the manuscript.

## Funding

This work was supported by Wroclaw Medical University statutory grant SUBZ.C200.22.067.

## Conflict of interest

The authors declare that the research was conducted in the absence of any commercial or financial relationships that could be construed as a potential conflict of interest.

## Publisher's note

All claims expressed in this article are solely those of the authors and do not necessarily represent those of their affiliated organizations, or those of the publisher, the editors and the reviewers. Any product that may be evaluated in this article, or claim that may be made by its manufacturer, is not guaranteed or endorsed by the publisher.
