# Supplementary material for: Short- and long-term outcome of allogeneic stem cell transplantation in infants: A single-center experience over 20 years
Source: Front Pediatr. 2022 Aug 22;10:956108. doi: 10.3389/fped.2022.956108 (PMC9441786; doi:10.3389/fped.2022.956108)
Supplement: Supplementary file 1 [file Data_Sheet_1.PDF]

Supplemental file

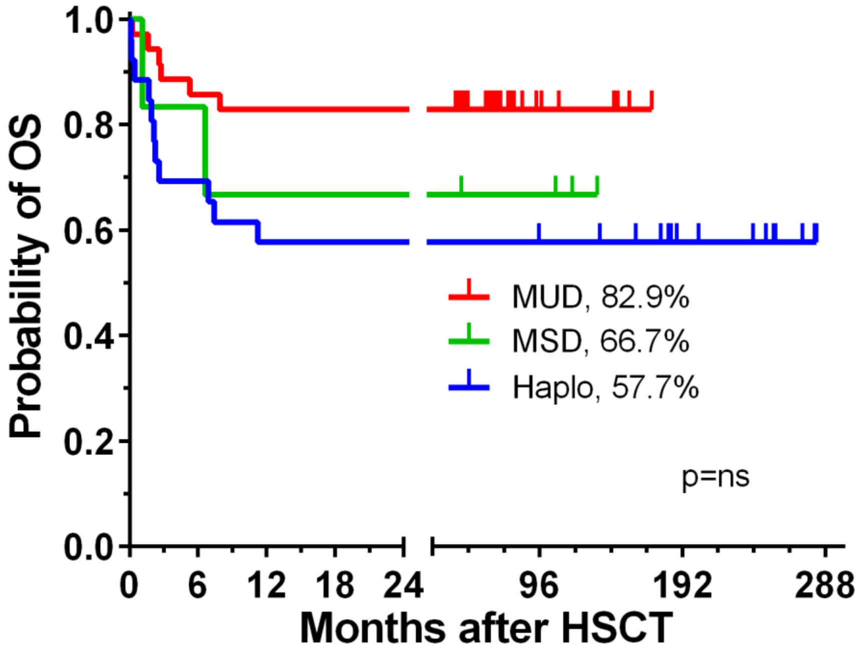

Supplemental figure 1. Overall survival of children transplanted from matched related (MSD), matched unrelated (MUD), and haploidentical (Haplo) donors.

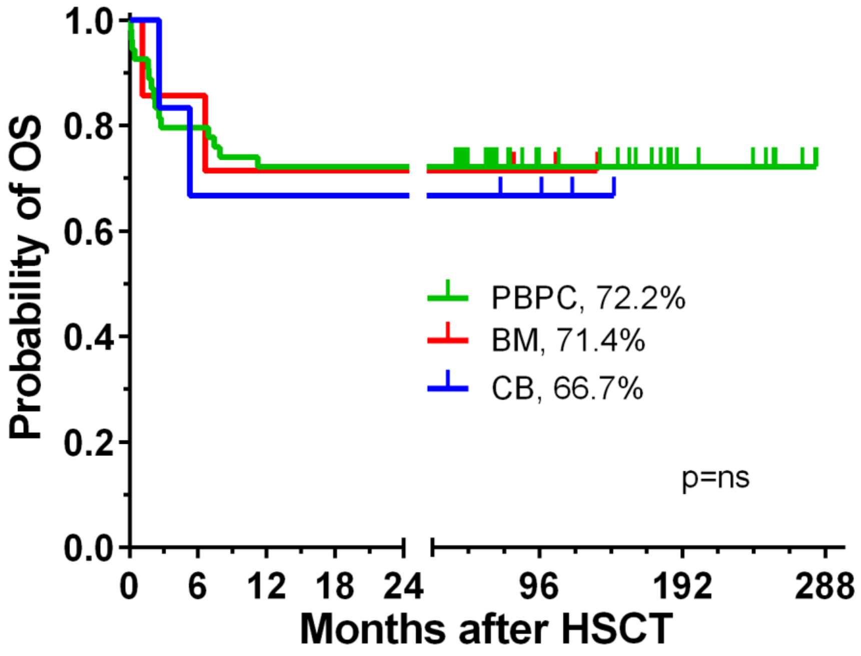

Supplemental figure 2. Overall survival in bone marrow (BM), peripheral blood progenitor cells (PBPC) and cord blood recipients (CB).

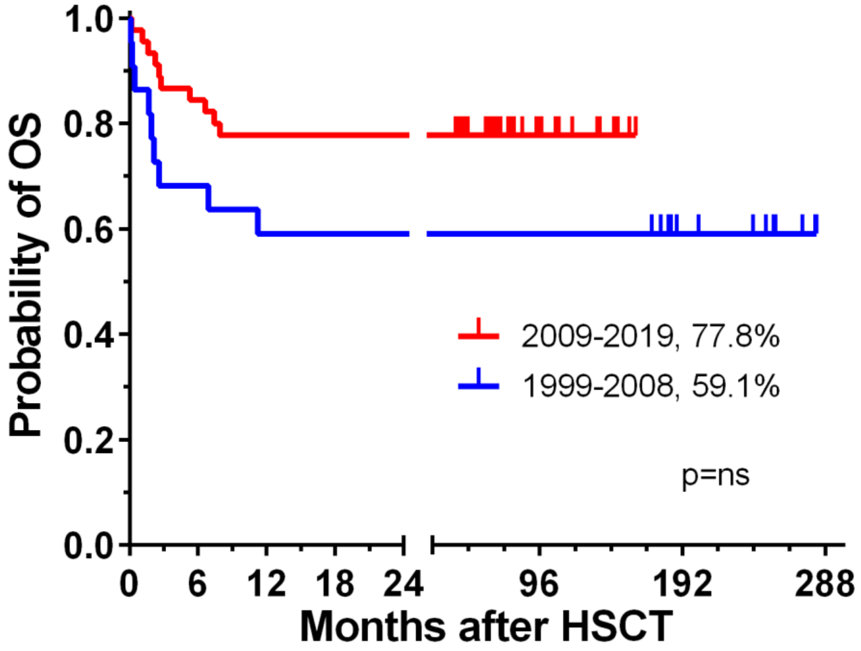

Supplemental figure 3. Overall survival of infants undergoing allo-HSCT in years 1999-2008 and 2009-2018.

Supplemental table 1. Analysis of risk factors for neurological complications

Risk factor	Type of risk factor	Complication present	Complication absent	Fisher exact test p-value
Underlying disease	malignant	1	14	1.0
	nonmalignant	4	48	
Type of donor	MSD/MUD	5	36	0.1479
	haplo	0	26	
Conditioning	Busulfan	2	38	0.3645
	Treosulfan	3	22	
Gender	Male	4	46	1.0
	Female	1	16	
Age at HSCT	0-5 months	1	16	1.0
	6-12 months	4	46	

Supplemental table 2. Analysis of risk factors for ocular complications

Risk factor	Type of risk factor	Complication present	Complication absent	Fisher exact test p-value
Underlying disease	malignant	4	11	1.0
	nonmalignant	14	38	
Type of donor	MSD/MUD	13	28	0.3969
	haplo	5	21	
Conditioning	Busulfan	10	30	0.7808
	Treosulfan	7	18	
Gender	Male	15	35	0.5272
	Female	3	14	
Age at HSCT	0-5 months	4	13	1.0
	6-12 months	14	36	

Supplemental table 3. Comparison of allo-HSCT outcome between periods 1999-2008 and 2009-2019.

Risk factor		1999-2008			2009-2019			Period comparision p-value
		number	pOS	p-value	number	pOS	p-value	
Gender	Male	17	0.64705	0.79680	33	0.84848	0.17691	0.07811
	Female	5	0.6		12	0.58333		0.69402
Primary disease	malignant	6	0.5	0.62749	9	0.88889	0.48586	0.13628
	non-malignant	16	0.6875		36	0.75		0.31876
Type of donor	MSD	0	-	0.60040	6	0.66667	0.05990	-
	MUD	2	0.5		33	0.84848		0.11519
	Haplo	20	0.65		6	0.5		0.62195
Stem cell source	BM	0	-	-	7	0.71429	0.81540	-
	PBPC	22	0.63636		32	0.8125		0.09887
	CBT	-	-		6	0.66667		-
Conditioning	busulfan	19	0.57895	0.35812	21	0.71429	0.66055	0.20524
	treosulfan	3	1		22	0.81818		0.63841
	other	0	-		2	1		-
Graft engineering	CD34 enrichment	16	0.625	0.70745	0	-	0.04749*	-
	T cell depletion	4	0.75		6	0.5		0.56705
	none	2	0.5		39	0.82051		0.16635

Statistically significant differences are marked with asterisk (‘*’).

Supplemental table 4. Impact of haploidentical graft engineering methods on the allo-HSCT outcome.

	CD34 enrichment (n=16)	T-cell depletion: 3/19 depletion or alpha-beta depletion (n=10)	p value
OS	0.5625	0.6	0.8590
EFS	0.3125	0.6	0.1541
TRM	0.3750	0.4	1.0
Graft failure	0.3125	0.1	0.3524
Second allo-HSCT	0.3750	0.0	0.0532
